# Early Successional Microhabitats Allow the Persistence of Endangered Plants in Coastal Sand Dunes

**DOI:** 10.1371/journal.pone.0119567

**Published:** 2015-04-02

**Authors:** Eleanor A. Pardini, Kyle E. Vickstrom, Tiffany M. Knight

**Affiliations:** Department of Biology, Washington University in St. Louis, St. Louis, Missouri, United States of America; Beijing Forestry University, CHINA

## Abstract

Many species are adapted to disturbance and occur within dynamic, mosaic landscapes that contain early and late successional microhabitats. Human modification of disturbance regimes alters the availability of microhabitats and may affect the viability of species in these ecosystems. Because restoring historical disturbance regimes is typically expensive and requires action at large spatial scales, such restoration projects must be justified by linking the persistence of species with successional microhabitats. Coastal sand dune ecosystems worldwide are characterized by their endemic biodiversity and frequent disturbance. Dune-stabilizing invasive plants alter successional dynamics and may threaten species in these ecosystems. We examined the distribution and population dynamics of two federally endangered plant species, the annual *Layia carnosa* and the perennial *Lupinus tidestromii*, within a dune ecosystem in northern California, USA. We parameterized a matrix population model for *L*. *tidestromii* and examined the magnitude by which the successional stage of the habitat (early or late) influenced population dynamics. Both species had higher frequencies and *L*. *tidestromii* had higher frequency of seedlings in early successional habitats. *Lupinus tidestromii* plants in early successional microhabitats had higher projected rates of population growth than those associated with stabilized, late successional habitats, due primarily to higher rates of recruitment in early successional microhabitats. These results support the idea that restoration of disturbance is critical in historically dynamic landscapes. Our results suggest that large-scale restorations are necessary to allow persistence of the endemic plant species that characterize these ecosystems.

## Introduction

Rare species with limited distributions are often those that have evolved to take advantage of spatially restricted, transient disturbances, which characterize a large number of ecosystems [1]. Human activities have dramatically altered many historical disturbance regimes. For example, dams and water control have limited periodic stream flooding, and thus negatively affected the floodplain species that are dependent on regular flood intervals [2]. Fire suppression has limited the ability of fire-dependent species to persist in their native ecosystems [3]. Invasive species have also dramatically transformed disturbance regimes with wide-ranging effects from altering fire frequency and intensity to changing soil disturbance patterns [4]. Invasive species change many biotic and abiotic factors simultaneously, so a deeper understanding of the ecological factors that limit population growth of disturbance-adapted species would allow for more precise conservation planning [5]. For example, is removing the invasive species and restoring the natural disturbance regime enough to increase populations of rare species, or must other factors altered by the invasion (e.g., changes in soil nutrients or microbial communities) also be managed?

The relationship between the successional state of the habitat and population dynamics of rare species is best understood by studies that consider multiple habitat types and integrate demographic data across the life cycle of the species. In an exemplary study, Smith and colleagues [6] found that the endangered floodplain plant *Boltonia decurrens* shifts its demography towards an annual life cycle and has much higher population growth rates in recently flooded areas, relative to more stabilized environments. Similar demographic studies have documented the importance of successional state or time since disturbance to plant population dynamics in systems that are disturbed by fire, [7,8], grazing [9,10] and hurricanes [11]. One important application of these studies is that they have been used to determine the frequency of disturbance necessary for species persistence.

Worldwide, coastal sand dunes are important for coastal defense against erosion, recreation, ecotourism, and as reservoirs of biodiversity [12]. They are a classic example of a dynamic ecosystem defined by disturbance and successional processes [13]. They consist of a mosaic of open and stabilized microhabitats created by geological processes and wind movement. Coastal sand dunes worldwide contain endemic plant species adapted to the frequent disturbance and unique edaphic conditions present, such as sand burial, moving substrate, low soil nutrients and moisture, and strong, salty winds [14,15]. Sand burial is a particularly important factor affecting plant distribution and vital rates [16][16–18]. Despite a rich history of research on dunes, little is known about the link between dune dynamics and the population-level processes of plants endemic to the ecosystem.

Coastal dune ecosystems are threatened worldwide due to human reduction of land area and modification of their disturbance regimes. Coastal dunes are attractive locations for human settlement and recreation. For example, nearly 75% of Mediterranean coastal dunes have been lost in the last 30 years [19] and in New Zealand, coastal sand dunes have declined from 129,000 ha to 39,000 ha (~70% of the habitat lost) over the past 100 years due to land conversion and erosion [20]. Similar losses are observed in North America, Australia, Europe and Asia [21]. Coastal dunes are highly susceptible to biological invasions, especially by plants intentionally introduced for dune stabilization. For example, *Rosa rugosa* (Japanese rose) shades native vegetation and alters soil nutrients in dunes throughout Europe as well as the northeastern U.S. [22]. Likewise, in western North America and Australia, *Ammophila arenaria* (European beachgrass) displaces native species, changes dune topology and stabilizes dunes [15,20,23,24]. Both invaders are difficult and expensive to manage because of deep underground clonal structures that re-sprout after they are disturbed by fire, herbicide, or manual removal [23,25,26].

The California Floristic Province along the western coast of North America is recognized as a global biodiversity hotspot known for its high plant endemism [27]. Sand dunes in this area have declined due to land modification and invasive plants, most notably the grass *Ammophila arenaria* [15,23]. One way in which *A*. *arenaria* threatens native ecosystems is by creating a high foredune and reducing interior sand movement, which reduces the frequency of blowouts that lead to ephemeral early successional habitats [24]. *Ammophila arenaria* is implicated in the decline of plant diversity [28,29], including six federally endangered plant species [30], and decline of the federally threatened Western snowy plover [31]. However, to date, no study has rigorously assessed the role of the successional dynamics in the decline of these rare species.

In this study, we examined dune plant communities at Point Reyes National Seashore (PRNS) in northern California, USA. The sand dunes of PRNS house a large proportion of the ranges of several rare plant species, but have become heavily dominated by *A*. *arenaria*, which has dramatically reduced the availability of early successional habitat within the dunes. We documented differences in community composition between early and late successional habitats. We quantified differences in the abundance of two federally endangered plants (*Lupinus tidestromii* and *Layia carnosa*) and in the stage structure of *L*. *tidestromii* between different successional microhabitats. We used three years of demographic data to parameterize a matrix population model of *Lupinus tidestromii* in early and late successional microsites. We used a life table response experiment (LTRE) to understand the role of each demographic parameter in causing the observed shift in population growth rate between these microhabitats.

## Materials and Methods

### Study area

This study was conducted on the dunes at Abbotts Lagoon within PRNS (Marin County, California). The area experiences a Mediterranean climate moderated by maritime influences, which includes short, wet winters, windy springs, and long, dry summers [32]. Dunes at PRNS consist of patches of remnant, native dune that are surrounded by introduced European beachgrass (*Ammophila arenaria*) and iceplant (*Carpobrotus* spp.). The 20 ha remnant dune immediately west of Abbotts Lagoon has been surrounded on all other sides by *A*. *arenaria*, which was introduced some time after the 1940s [33]. The remnant dune area is characterized by a high foredune with interior dunes that consist of early, middle, and late successional habitats and is home to high plant diversity. In remnant dune patches, some sand movement in the direction of NW prevailing wind is typical [34], but the high foredune has likely altered historical disturbance regimes and reduced interior sand. Between January and July 2011 PRNS conducted a large-scale dune restoration project at Abbotts Lagoon and successfully removed 32 ha of *A*. *arenaria* from a 77 ha area surrounding the remnant native dune patch by mechanical removal and hand-pulling [33].

### Study species

*Lupinus tidestromii* (Fabaceae) is a low, herbaceous, perennial plant endemic to coastal dunes in Sonoma, Marin, and Monterey counties in California. It is federally endangered and now occurs in 15 locations, 9 of which are located within PRNS [35]. Abbotts Lagoon is home to the largest extant population of *L*. *tidestromii* which has ranged in size from ~90,000 to 176,000 individuals between 2001 to 2012 [33]. Across its range, *L*. *tidestromii* is threatened by habitat loss, trampling by visitors and cattle, hybridization, direct competition with invasive plants, and elevated levels of seed predation in the presence of *A*. *arenaria* [36,37]. The primary threats at Abbotts Lagoon are from *A*. *arenaria*, which competes with native vegetation, houses elevated levels of native seed predators [34,36,38], and stabilizes native dunes, reducing the amount of suitable early successional habitat that appears to favor seedling establishment [33].

*Layia carnosa* (Asteraceae) is a succulent, winter annual that is endemic to coastal dunes in Humboldt, Marin, Monterey, and Santa Barbara counties in California. It is federally endangered and is threatened by land development, encroachment by invasive plants, and recreational habitat use [37]. *Layia carnosa* recruits readily in open sand with sparse vegetation, and population dynamics appear to be tied to dune blowouts [37]. At PRNS, the most significant threat is thought to be reduction of early successional habitat due to dune stabilization by *A*. *arenaria*, but there is little quantitative data to support this.

### Community composition across habitats of different successional stages

To describe community composition of early, middle, and late successional habitats at Abbott’s Lagoon, in 2008 we sampled vegetation in 180 1-m^2^ quadrats across the northern half of the population. We sampled 5 1-m^2^ quadrats at each of 36 sites along two N-S transects; sites were separated by at least 50 m. At each site, the 5 quadrats were separated from one another by 5 m. In each quadrat, we identified all herbaceous forbs to species and placed them in one of nine percent cover categories (0%; <1%, 1–5%, 6–15%, 16–25%, 26–50%, 51–75%, 76–90%, 91–100%). We also estimated the percent cover of bare sand in the quadrat using the same categories.

For analysis, quadrats were categorized as early, mid or late successional stages based on their percent cover of bare sand (>75%, 25–75%, <25% bare sand, respectively). To get a single value of percent cover for each species and quadrat, we used the midpoint of the percent cover category. We averaged the percent cover of each species across quadrats within a sample site that were in the same successional stage, creating a data set of 69 sites for analysis (because some sites contained multiple successional stages). We calculated similarity in community composition across sites using a Bray-Curtis metric. We used Permutational Multivariate Analysis of Variance (PERMANOVA) in PRIMER-E to test whether sites within a successional stage category are more similar to each other than they are to those in other categories [39].

### Frequency of endangered plants and stage structure of *L*. *tidestromii*

To examine the frequency and stage structure of *L*. *tidestromii* and *L*. *carnosa* across the entire native dune area, in 2012 we surveyed vegetation in 207 1-m^2^ vegetation plots on a 50 m grid system that was placed on a randomly chosen start point. In each plot, we recorded density of *L*. *carnosa* and *L*. *tidestromii*. The successional stage of each plot was scored as early, middle, or late, based on the percent of the plot area occupied by bare sand (≥70% bare sand for early; 35%-90% for mid; 0%-40% for late) and by the identity of occurring plants (e.g., presence of stabilizing grass species). For analysis, to ensure the frequency of plants in the early, mid, and late successional stage plots was reflective of pre-restoration vegetation frequencies, we included only those 145 plots that were not blown over by sand movement resulting from the 2011 dune restoration. Of the 145 plots included in the analyses, 22 (15%) were classified as early (mean 84.4% bare ground), 98 (68%) were classified as mid (mean 60% bare ground), and 17% were classified as late (mean 18.8% bare ground). We calculated the proportion of plots within each type of microhabitat that contained *L*. *tidestromii* or *L*. *carnosa*. For *L*. *tidestromii*, we further calculated the proportion of individuals in each of three stage classes (seedling, non-reproductive, and reproductive) found within each successional microhabitat type.

### Matrix population model for *L*. *tidestromii*

**Fig 1:**
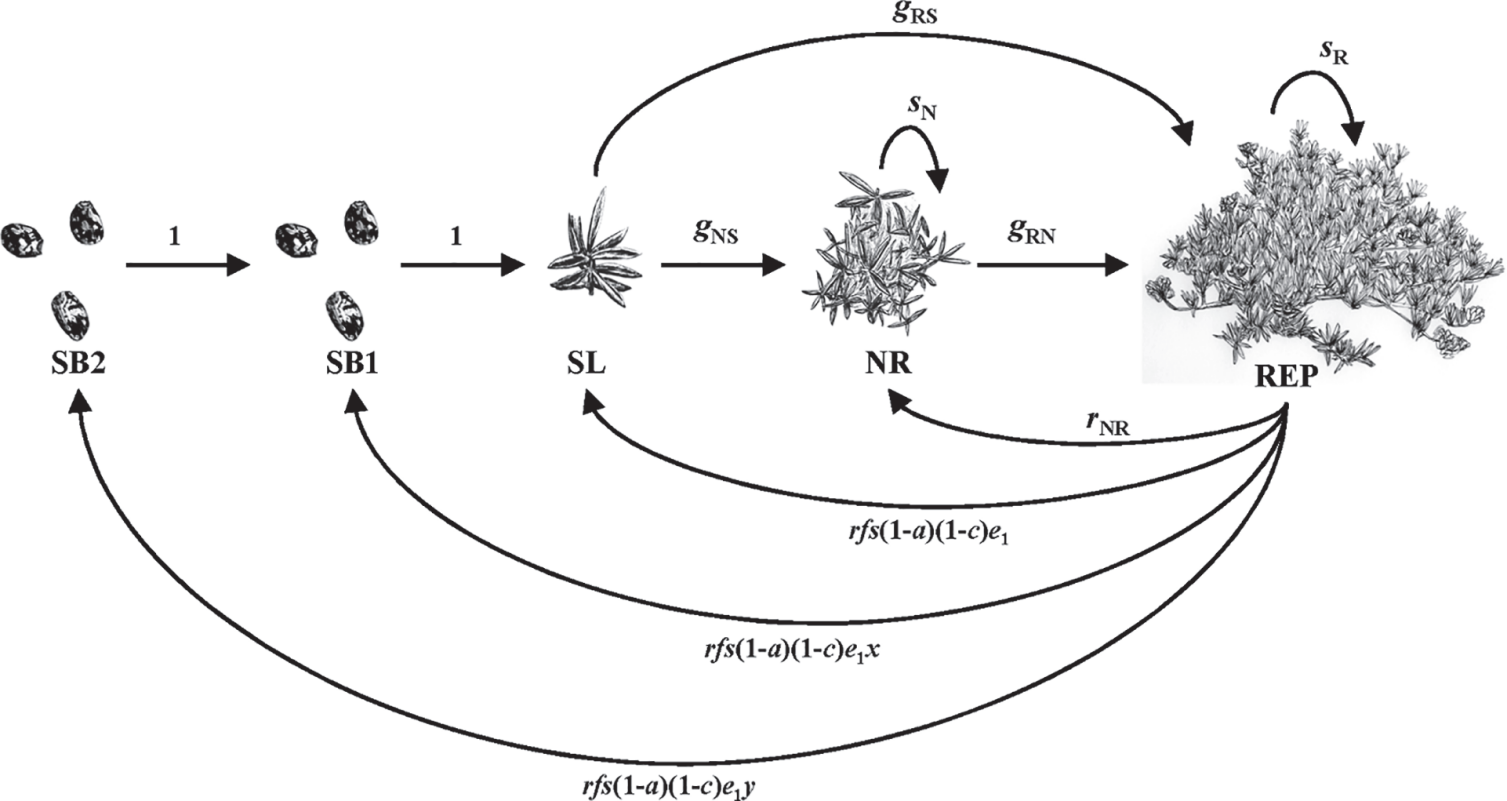
Life cycle diagram for *Lupinus tidestromii* representing annual transitions among five life stages. Key to abbreviations: SB2, seeds in the seed bank that will germinate in two years; SB1, seeds in the seed bank that will germinate in one year; SL, seedling; NR, non-reproductive; REP, reproductive. Illustrations by Patrick Hanley.

We used matrix population models to project the effects of microhabitat on the population dynamics of *L*. *tidestromii*. *Lupinus tidestromii* is well-described by a stage-structured model that includes five stage classes: seeds in a seed bank that will germinate after three winters (SB2), seeds in a seed bank that will germinate after two winters (SB1), seedlings (SL), non-reproductive plants (NR), and reproductive plants (REP, see Fig. 1). The results presented here are part of a larger study we are conducting on *L*. *tidestromii*. Our larger study includes demographic and genetic data collection for additional plants, years (2005-present at Abbotts Lagoon), and populations to address a broad set of questions. In 2005 we began monitoring plants along 3 transects located in the northern half of the site. In 2008 we expanded our sampling to include clusters of plants located along two N-S transects that spanned the entire length of the population. Clusters were chosen to include all stage classes of *L*. *tidestromii*. Plants were chosen within each cluster to provide adequate sample size of each stage class. When plants within a stage class were overrepresented within the cluster, individuals were haphazardly chosen for inclusion in the study. Seedlings are more frequent in early successional microhabitats, thus our efforts to include clusters containing all stage classes resulted in underrepresentation of individuals in late successional habitat, especially for the seedling stage. In 2010, we expanded our sampling to include more clusters of individuals in mid and late successional habitat and began recording the microhabitat of the surrounding 1-m^2^ for each tagged plant. We scored the microhabitat as early, mid, or late by observation of the amount of bare sand, amount of vegetation, and type of vegetation present. Late microhabitats contained between 0–40% bare ground and were dominated by the grass *Poa douglasii* and/or shrubs such as *Ericameria ericoides*; Mid-successional microhabitats contained between 35–75% bare ground and were dominated by the grass *Poa douglasii* and/or perennial forbs such as *Abronia latifolia*; Early microhabitats had between 70–100% bare ground and tended to contain less grass cover and no shrubs. To address the specific questions here, we constructed models from demographic data collected from 2010–2012 during which we recorded microhabitat of all plants.

Vital rates of seedlings, non-reproductive plants and reproductive plants were quantified by observing survival and fertility of tagged plants in June of each year. Numbered aluminum tags were attached to the basal stem of or staked near plants, and GPS and a metal detector were used to relocate plants each year to obtain estimates for growth, stasis, and regression (*g*_*RS*_, *g*_*NS*_, *g*_*RN*_, *s*_*N*_, *s*_*R*_, *r*_*NR*_, Fig. 1). In each year we tagged at least 50 new seedlings and additional non-reproductive plants to supplement sample sizes reduced by growth or mortality.

For *L*. *tidestromii*, we estimate fertility as a product of the average number of racemes per plant (*r*), average number of fruits per raceme (*f*), and the average number of seeds per fruit (*s*). Transition from the reproductive class to the seed and seedling classes also depends on rates of fruit abortion (*a*) and pre-dispersal seed consumption (*c*). We quantified *r* by counting racemes on tagged reproductive plants (N = 121 reproductive plants in 2010 and 168 in 2011); *r* varies across years and microhabitats. We quantified *s* by randomly collecting 23, 3, 15, and 39 fruits in 2005, 2008, 2009, and 2010 at this field site and counting the number of seeds per fruit. We calculated the mean number of seeds per fruit across all fruits and years (n = 80) and use this as a constant in the model (we assume that *s* does not change across years or microhabitats). To quantify *a*, *c*, and *f*, we followed the fates of marked racemes on each reproductive plant each year. Following marked racemes was not possible in 2010 because reproduction of *L*. *tidestromii* was unusually low. In 2010, we recorded the number of racemes that were dehisced/would dehisce soon, were aborted, or had been completely consumed (evidenced by a diagonal clip on a stalk and missing inflorescence) on 131 reproductive plants at one point in time during our demographic census of tagged plants (June 7—June 18). In 2011 we tied colored yarn to all racemes (up to 16 per plant, mean 3.73 yarns per plant) on 148 reproductive plants between June 6-June 14 and we returned to these racemes every 2–3 weeks until all fruits had dehisced (June 14-July 7) to record if the raceme produced fruits, was aborted, or was consumed. To calculate *a* and *c*, we summed the number of aborted racemes or consumed racemes and divided by the sum of the total number of observed racemes. To calculate *f* (estimate available for 2011 only), we averaged the number of fruits across all racemes for each plant and then averaged across plants to produce the population-level value of *f*. We assume that *a* and *c* vary across years but not habitats and *f* is constant across years and microhabitats.

Our model requires understanding how microhabitats affect recruitment and how recruitment changes over time for seeds in the seed bank. We estimated microhabitat-specific recruitment (*e*_1_) for the transition from reproductive plants to seedlings from a set of paired seedling quadrats that were established in early and late microhabitats in 2011–2012 (note that the parameter *e*_1_ includes mortality due to post-dispersal seed predation). To test for differences in *e*_1_ between microhabitats we used a randomization test with 10,000 replicates. We estimated how recruitment changes over time from seed baskets that were established in 2008 and in which germination was observed in 2009–2011. For the reproductive to seed bank transitions, we adjust the *e*_1_ values by a proportion *x* or *y* determined from long-term seed baskets (methods detailed in Supporting Information S1 File); *x* is the ratio of seeds that germinated after two winters to the number of seeds that germinated after one winter, and *y* is the ratio of seeds that germinated after three winters to the number of seeds that germinated after one winter (Fig. 1; S1 File).

To quantify the effect of microhabitat on the population dynamics of *L*. *tidestromii*, we projected deterministic population growth rate (λ) for each transition year (2010–2011; 2011–2012) and microhabitat (early; late) using a matrix population model [40]. We calculated 95% confidence intervals around vital rates and population projections using 1,000 bootstrapped simulations. Specifically, we sampled five datasets with replacement to generate bootstrap datasets, calculated vital rates from each bootstrap dataset, and then projected population growth rates. The datasets used for bootstrapping were: (1) individuals in the demography dataset for parameters *g*_*RS*_, *g*_*NS*_, *g*_*RN*_, *s*_*N*_, *s*_*R*_, *r*_*NR*_, and *r*, (2) individuals in the raceme dataset for parameters *f*, *a*, and *c*, (3) fruits in the fruit dataset for parameter *s*, (4) seedling quadrats to generate *e*_1_, and (5) seed baskets to generate *x* and *y*. Analyses were performed in MATLAB (Math Works 2010).

We made the following assumptions in our model. First, we assumed that some fertility parameters are constant among habitats, years or both. We have observed that fertility in this population varies largely in the number of racemes, rather than in the number of seeds per fruit or fruits per raceme. Thus we measured microhabitat-specific values of *r* but we hold *a*, *c*, and *f* constant across microhabitats. Second, we assume that seeds do not persist in the seed bank for more than two years. This assumption matches our observations in the seed baskets. Seedling recruitment was observed in 2009–2011, but not in 2012. This assumption is reasonable in the current habitat, in which large disturbances that bring deeply buried seeds to the surface are nonexistent due to the stabilization of sand by *A*. *arenaria*. However, we note that *L*. *tidestromii* has a seed coat that should allow for long-term dormancy. Third, we assume that parameters are density-independent. It is well known that seed consumption rates (i.e., *c* in our model) respond in a compensatory manor to plant and seed densities. We use a simple, density-independent model because our main objective is to examine potential differences among successional microhabitats in mean population fitness, not to forecast population size.

### Life table response experiment

Nine matrix elements varied between plants in early and late successional habitats. We used a life table response experiment (LTRE) to determine how each of these contributed to the overall difference in λ observed across habitat types in each of our study years [40]. The contribution of each element is determined by the product of the difference in the element between early and late successional habitats (*a*_*ij*_^*early*^—*a*_*ij*_^*late*^) and the sensitivity of λ to changes in each matrix element (*s*_*ij*_). The sensitivities were calculated from a matrix that was the mean of early and late successional habitat matrices in each year. Analyses were performed in MATLAB [41].

## Results

### Community composition across habitats of different successional stages

**Fig 2:**
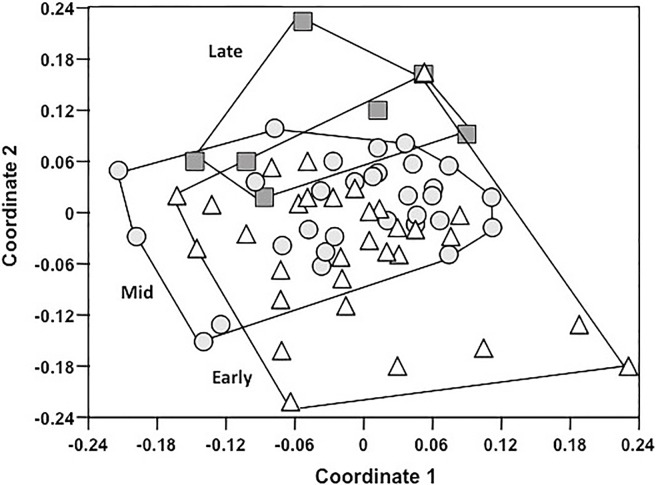
Vegetation community composition of successional microhabitats at Abbotts Lagoon. Non-metric multidimensional scaling ordination plots of community composition in two-dimensional space. Each point represents the composition of herbaceous plants in a site in multidimensional space, and the distance between any two points represents the difference between those two sites according to a modified Raup-Crick metric. Sites that are closer together are more similar in community composition. Open triangles represent the early successional sites, gray circles represent mid successional sites and gray squares represent late successional sites. Lines represent the minimum convex hulls around the data.

Successional stage significantly affected plant community composition (PERMANOVA *SS* = 21.26, *F* = 2.51, *P* = 0.001; Fig. 2), and pairwise differences between successional stage categories showed that all were distinct from each other in composition except for the mid and late successional stages (Early vs. Mid *P* = 0.009, Early vs. Late *P* = 0.049, Mid vs. Late *P* = 0.11). *Lupinus tidestromii* was the most abundant species on early successional plots, whereas *Abronia latifolia* dominated late successional plots.

### Frequency of endangered plants and stage structure of *L*. *tidestromii*

**Fig 3:**
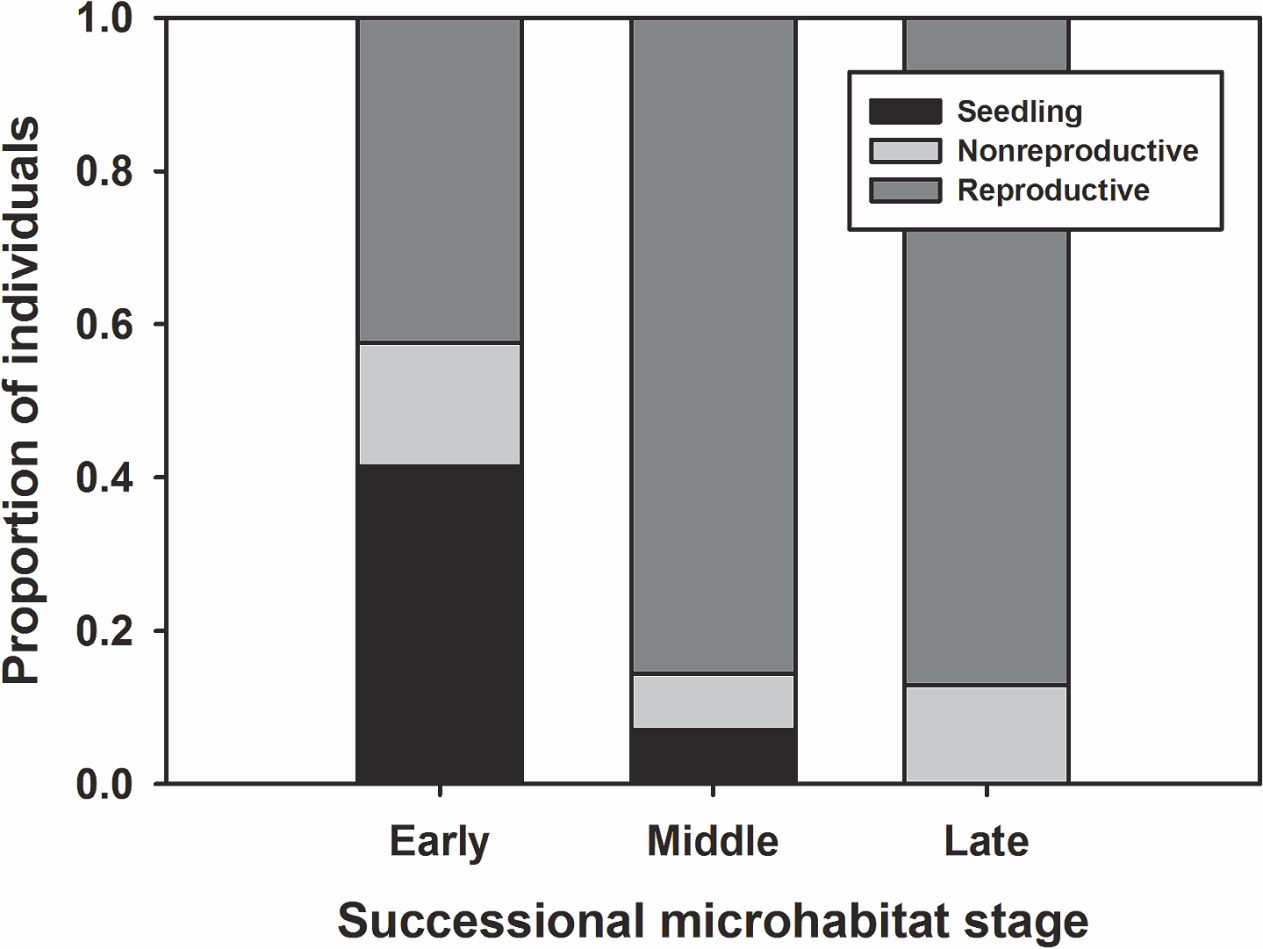
Stage structure of *Lupinus tidestromii* in three successional microhabitats at Abbotts Lagoon. Stacked bars indicate the proportion of *Lupinus tidestromii* individuals in each of three stage classes (seedling, non-reproductive, and reproductive) found in each of three successional microhabitats at Abbotts Lagoon. Individuals were pooled across plots within each of the three habitat types. Within 145 vegetation plots, there were 198 plants in 22 early plots, 382 plants in 98 mid plots, and 31 plants in 25 late plots; in total, 109 seedlings, 64 non-reproductive plants, and 438 adult plants were found.

The frequency of plots containing *L*. *tidestromii* and *L*. *carnosa* differed significantly among successional microhabitats, with the highest frequency of occurrence in early plots and the lowest in late plots (Table 1). In 112 plots where *L*. *tidestromii* occurred, the stage structure differed among successional microhabitats. Seedling occurrence differed significantly among habitat types (χ2 = 36.07, df = 2, *P*<0.001): 86% of early plots contained seedlings while no late plots contained seedlings (Fig. 3). Most plots (102 of 112) contained adult *L*. *tidestromii*, but the occurrence of adults was lowest among early plots (Fig. 3).

**Table 1:** Frequency of *Lupinus tidestromii* and *Layia carnosa* in early, mid, and late successional microhabitats at Abbotts Lagoon.

	Plots(n)	*L*. *tidestromii* n (%)	*L*. *carnosa* n (%)
Early	22	22 (100%)	11 (50%)
Mid	98	79 (81%)	13 (13%)
Late	25	11 (44%)	0 (0%)
χ^2^		22.8302	23.5451^a^
P-value		<0.0001	<0.0001

Indicated are the number of plots sampled and the number (n) and percentage of those plots that contained *L*. *tidestromii* and *L*. *carnosa* individuals.

^a^33% of the cells have expected counts less than 5; chi-square may not be a valid test.

### Matrix population model for *L*. *tidestromii*

**Fig 4:**
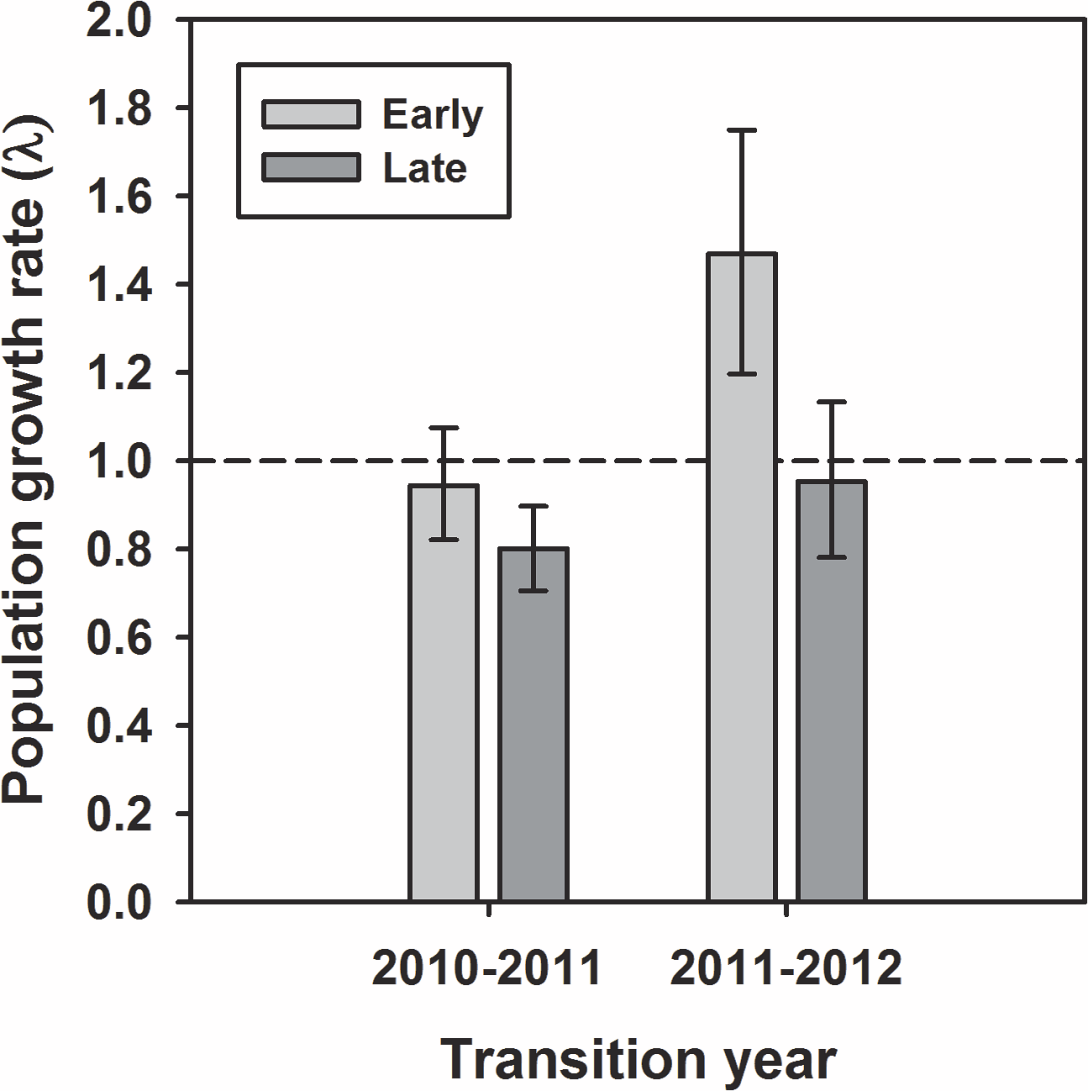
Population growth rate of *Lupinus tidestromii* in different successional microhabitats. Deterministic matrix model projections of population growth rate (λ) for *Lupinus tidestromii* at Abbotts Lagoon are higher in early microhabitats in 2010 and 2011. We present observed values and 95% confidence intervals from 1,000 bootstrap estimates.

The stage transitions of the seedling, non-reproductive, and reproductive plants did not vary in a consistent manner between microhabitats. For example, there were more seedlings that became non-reproductive plants in the early compared to the late successional habitat in 2011–2012, but this was not the case in the 2010–2011 (Table 2). In 2010 compared to 2011, plants produced fewer racemes and those racemes were more likely to abort their fruits or be consumed by pre-dispersal seed predators. Recruitment (*e*_1_) was significantly higher (randomization test, *P* = 0.0023) in early compared to late successional habitats in both years (Table 3). As a result, projected rates of population growth (λ) were higher in 2011 compared to 2010 and in both years, plants in early successional microhabitats had a higher λ than those in late successional microhabitats (Fig. 4).

**Table 2:** Average transition matrices (with 95% bootstrapped confidence intervals and sample sizes for growth, survival, and regression transitions) for *Lupinus tidestromii* in early and late successional microhabitat at Abbotts Lagoon.

*L*. *tidestromii*	*Plant stage at year t*				
*Stage at year t+1*	*SB2*	*SB1*	*SL*	*NR*	*REP*
2010–2011 Early					
SB2	0	0	0	0	0.0039 (0.0000–0.0127)
SB1	1	0	0	0	0.1974 (0.0571–0.5646)
SL	0	1	0	0	0.2409 (0.1123–0.3995)
NR	0	0	0.5583 (0.4667–0.6417, 120)	0.2561 (0.1585–0.3537, 82)	0.0877 (0.0351–0.1579, 57)
REP	0	0	0.0750 (0.0333–0.1250, 120)	0.5000 (0.3902–0.6098, 82)	0.6491 (0.5088–0.7544, 57)
2010–2011 Late					
SB2	0	0	0	0	0.0005 (0.0000–0.0018)
SB1	1	0	0	0	0.0235 (0.0044–0.0740)
SL	0	1	0	0	0.0287 (0.0065–0.0561)
NR	0	0	0.5294 (0.3922–0.6667, 51)	0.2174 (0.1087–0.3261, 46)	0.0968 (0.0323–0.1774, 62)
REP	0	0	0.2745 (0.1569–0.4118, 51)	0.5870 (0.4348–0.7174, 46)	0.6452 (0.5161–0.7581, 62)
2011–2012 Early					
SB2	0	0	0	0	0.0190 (0.0000–0.0681)
SB1	1	0	0	0	0.9497 (0.4209–1.6198)
SL	0	1	0	0	1.1586 (0.8945–1.4678)
NR	0	0	0.4286 (0.2857–0.5714, 42)	0.2029 (0.1159–0.3043, 69)	0.0392 (0.0000–0.0980, 51)
REP	0	0	0.4286 (0.2857–0.5952, 42)	0.6087 (0.4928–0.7246, 69)	0.6667 (0.5294–0.8039, 51)
2011–2012 Late					
SB2	0	0	0	0	0.0028 (0.0000–0.0098)
SB1	1	0	0	0	0.1397 (0.0248–0.4678)
SL	0	1	0	0	0.1704 (0.0.0410–0.3348)
NR	0	0	0.2530 (0.1687–0.3494, 83)	0.1220 (0.0244–0.2439, 41)	0.0274 (0.0000–0.0685, 73)
REP	0	0	0.4819 (0.3735–0.5783, 83)	0.6829 (0.5366–0.8293, 41)	0.6986 (0.6027–0.7945, 73)

Key to abbreviations: SB2, seeds in the seed bank that will germinate in two years; SB1, seeds in the seed bank that will germinate in one year; SL, seedling; NR, non-reproductive; REP, reproductive.

**Table 3:** Average fertility values (with 95% bootstrapped confidence intervals and sample sizes) for *Lupinus tidestromii* in early and late successional microhabitats at Abbotts Lagoon.

*Parameter*	*2010*–*2011*	*2011*–*2012*
*r, early*	6.4746 (5.0508–8.0847, 59)	8.4368 (6.8276–10.3103, 87)
*r, late*	5.9355 (4.9839–6.9194, 62)	9.5432 (7.7160–11.7901, 81)
*f* ^a^	5.1847 (4.7519–5.6348, 135)	5.1847 (4.7626–5.5993, 135)
*s* ^b^	3.3756 (3.0585–3.7059, 80)	3.3756 (3.0454–3.7464, 80)
*a*	0.4517 (0.3985–0.5026, 131)	0.1431 (0.1017–0.1881, 148)
*c*	0.6178 (0.5559–0.6682, 131)	0.0973 (0.0511–0.1538, 148)
*x* ^b^	0.8197 (0.3137–2.1707, 15)	0.8197 (0.3301–2.2286, 15)
*y* ^b^	0.0164 (0.0000–0.0476, 15)	0.0164 (0.0000–0.0476, 15)
*e*_1_, early^c^	0.0101 (0.0052–0.0155, 10)	0.0101 (0.0049–0.0161, 10)
*e*_1_, late^c^	0.0013 (0.0003–0.0024, 9)	0.0013 (0.0003–0.0024, 9)

^a^ Data to estimate *f* were only available in 2011, thus we use 2011 data for both years.

^b^ The parameters *s*, *x*, and *y* are constant across years and microhabitats.

^c^ The estimates for *e*_1_ differ by microhabitat but not by year

### Life table response experiment

LTREs revealed that early successional habitats result in a change in λ primarily by increasing the number of seedlings that recruit, either right away (REP to SL matrix element) or after spending one winter in the seed bank (REP to SB1 transition). These vital rates changed dramatically between early and late microhabitats, and were matrix elements with intermediate sensitivities (Table 4).

**Table 4:** Life table response experiment for *Lupinus tidestromii* populations in early versus late successional microhabitats at Abbotts Lagoon.

Year	Matrix element	*a*_*ij*_^early^	*a*_*ij*_^late^	*a*_*ij*_^early^−*a*_*ij*_^late^	*s*_*ij*_	Contribution
2010–2011	REP to SB2	0.0039	0.0005	0.0034	0.5280	0.0018
	REP to SB1	0.1974	0.0235	0.1739	0.4759	0.0828
	REP to SL	0.2409	0.0287	0.2122	0.4289	0.0910
	SL to NR	0.5583	0.5294	0.0289	0.1472	0.0043
	SL to REP	0.0750	0.2745	−0.1995	0.1800	−0.0359
	NR to NR	0.2561	0.2174	0.0387	0.1913	0.0074
	NR to REP	0.5000	0.5870	−0.0870	0.2340	−0.0204
	REP to NR	0.0877	0.0968	−0.0091	0.5103	−0.0046
	REP to REP	0.6491	0.6452	0.0039	0.6240	0.0024
2011–2012	REP to SB2	0.0190	0.0028	0.0162	0.1777	0.0029
	REP to SB	0.9497	0.1397	0.8100	0.2256	0.1827
	REP to SL	1.1586	0.1704	0.9882	0.2863	0.2829
	SL to NR	0.4286	0.2530	0.1756	0.2811	0.0494
	SL to REP	0.4286	0.4819	−0.0533	0.4817	−0.0257
	NR to NR	0.2029	0.1220	0.0809	0.0963	0.0078
	NR to REP	0.6087	0.6829	−0.0742	0.1650	−0.0122
	REP to NR	0.0392	0.0274	0.0118	0.3242	0.0038
	REP to REP	0.6667	0.6986	−0.0319	0.5556	−0.0177

Results are shown separately for year 2010–2011 and 2011–2012. Key to abbreviations: SB2, seeds in the seed bank that will germinate in two years; SB1, seeds in the seed bank that will germinate in one year; SL, seedling; NR, non-reproductive; REP, reproductive.

## Discussion

Our study is the first to rigorously assess the importance of early successional habitat for the abundance and demography of two endangered plant species, *L*. *carnosa* and *L*. *tidestromii*. Our surveys revealed that both of these endangered plant species were more frequent in early successional habitats. Our detailed demographic study of *L*. *tidestromii* revealed that plants in early successional microhabitats had higher projected rates of population growth than those associated with late successional, stabilized microhabitats, due primarily to higher rates of recruitment in early successional microhabitats.

This understanding of the reliance of rare plant species on early successional habitats is timely for management and restoration programs, because sand-stabilizing invasive plants are a pervasive problem in many coastal dune systems worldwide. Specifically, *A*. *arenaria* has invaded sand dune ecosystems across western North America, and it has fundamentally altered the topography, dynamics processes, and biodiversity of native plants and animals. Sand stabilization reduces interior sand movement and the frequency with which windstorms lead to blowouts that reset the successional trajectory by creating ephemeral early successional habitats [23,24]. *Ammophila arenaria* has been documented in western North America to alter plant distribution, reduce species richness, and reduce species evenness [28,29,34,42].

Dune stabilization likely threatens a variety of native plants and animals that are highly adapted to these dynamic systems. Western snowy plovers (*Charadrius alexandrinus nivosus*) select sites with open sand for their courtship and nesting [43]. The spread of *A*. *arenaria* in western North America has been implicated as a primary reason for the threatened federal status of these birds [31]. Both of our focal endangered plant species have traits that facilitate their survivorship and growth in early successional habitats in dynamic dune environments: *L*. *carnosa* has succulent, sticky leaves, wind dispersed seeds, and an annual life cycle; *L*. *tidestromii* has a long taproot, a prostrate habit, hairy leaves, and seed dormancy. Scarification of the seed coat is required for germination of *L*. *tidestromii* seeds, and thus, seeds can remain dormant in late-successional microhabitats until a disturbance brings seeds to the surface and scarifies them [37].

Our research suggests that current and future removal of intransigent *A*. *arenaria* from PRNS will likely lead to considerably higher population levels of *L*. *tidestromii*. While the rates of survival, growth, and fertility of established plants did not consistently differ between early and late successional microhabitats, recruitment of seedlings was 10-fold higher in early compared to late successional microhabitats. Our LTRE indicated that this difference contributed to the difference in population growth rate (λ) across microhabitats. For *L*. *tidestromii*, removal of *A*. *arenaria* should not only provide the early successional habitat that is critical for increased seedling recruitment rates, as we have shown here, but also provide a respite from heavy seed predation by *Peromyscus maniculatus* which utilize *A*. *arenaria* as refugia from which to forage [36].

Despite the clear benefits for these endangered species, removal of *A*. *arenaria* is exceptionally difficult due to its deep root system. For example, hand removal of the species often fails to remove deep root structures, and allows *A*. *arenaria* to re-sprout (Pickart 1997). Two methods that can successfully remove *A*. *arenaria* are the use of a combination of fire and herbicides because fire stimulates plant regrowth that readily absorbs herbicides [44] and the use of large machinery that can dig 1–3 meters into the ground and destroy the deep root structures of the plant [45].

A large-scale restoration project conducted between January and July 2011 at Abbotts Lagoon involved the removal of 32 ha of *A*. *arenaria* from a 77 ha area and landscape grading with heavy machinery to restore the topology of the dunes. There has been an immediate response of *L*. *tidestromii* to the dune restoration. Tens of thousands of seedling emerged in the open, bare sand, beginning in August 2011. By summer 2012, approximately 25% of these individuals were reproductive and set seed in their first year (W Johnson, S Minnick, & L Parsons, unpublished data). In the years following the restoration (2011–2014), seed predation levels were considerably lower than those levels observed in our 2005–2010 study years (EAP & TMK, unpublished data). These initial observations are consistent with the projected population response of this endangered species to early successional habitats made by our model.

## Conclusions

To our knowledge, ours is the first study to directly link plant demography to successional dynamics in sand dune ecosystems. Our results are consistent with results found in many other ecosystems subject to periodic disturbances and subsequent successional dynamics, such as those with fire [7,8], hurricanes [11], and floods [6]. These studies collectively show that the persistence of native species with traits that facilitate their use of early successional habitats is often linked to the maintenance of historical disturbance regimes. Our results are likely generalizable to other sand dune ecosystems. Many plant species endemic to sand dunes are adapted to tolerating the dynamic moving sand that historically characterized these ecosystems [14,15] and many of these plants are threatened by dune-stabilizing plant invaders that reduce the frequency and expanse of early successional microhabitats, such as *R*. *rugosa* [22] and *A*. *arenaria* [23]. Removal of these invaders with the purpose of restoring disturbance dynamics and mosaic landscapes should be a conservation priority.

## Supporting Information

S1 File Description of methods and data used to estimate seedling recruitment for *Lupinus tidestromii*. (DOCX)
