# Supplementary material for: Early Successional Microhabitats Allow the Persistence of Endangered Plants in Coastal Sand Dunes
Source: PLoS One. 2015 Apr 2;10(4):e0119567. doi: 10.1371/journal.pone.0119567 (PMC4383614; doi:10.1371/journal.pone.0119567)
Supplement: S1 File — (DOCX) [file pone.0119567.s001.docx]

**Supporting Information File S1: Description of methods and data used to estimate seedling recruitment for *Lupinus tidestromii*.**

**Seedling recruitment**

Our model requires understanding how microhabitats affect recruitment and how recruitment changes over time for seeds in the seed bank. We estimated the former from a set of paired seedling plots that were established in early and late microhabitats in 2011-2012. We estimated the latter from seed baskets that were established in 2008 and from which recruitment was observed in 2009-2011. Details of each of these experiments and results are provided below.

***Establishment of seedlings in early versus late successional microhabitats***

To measure differences in seedling recruitment rates we established seedling vegetation plots in early and late successional microhabitats. On July 18, 2011 we established 10 pairs of 2 m^2^ plots spaced out across the entire north to south extent of the Abbotts Lagoon dune area. Each pair contained two adjacent plots, with one in early and one in late successional microhabitat. Early habitat plots contained >90% bare sand with sparse vegetation and late microhabitat plots contained 15-50% bare sand and stabilizing vegetation such as *Poa douglasii*, *Lathyrus latifolius*, and *Abronia latifolia*. We selected plot areas that contained several reproductive plants that would contribute to seedling recruitment the following year, but that had few reproductive plants immediately adjacent to but outside the plot boundaries. In each plot we counted the number of reproductive *L. tidestromii*, including any plant whose basal stem was located within the plot margins. We assumed equivalent densities of seeds in the seedbank and recruitment of seeds from the seedbank in both early and late microhabitats.

On June 14, 2012, we returned to seedling plots and searched them exhaustively to count the number of new seedlings in the plot. Establishment measured at this point in time included seedlings that germinated and survived to the census point. To estimate the rate of establishment for the two types of plots, we first estimated seed rain within the plot based on the number of reproductive plants in the plot in 2011. Total seed rain was estimated as the product of the number of reproductive plants and the average number of seeds per plant (i.e. [(number of reproductive plants)*rfs*(1-*c*)(1-*a*)]) (Table A1). To estimate establishment rates, *e*_1_, we divided the number of seedlings observed in June 2012 by the estimated seed rain for each plot (Table A2). Mean establishment (*e*_1_) across plots was 0.0101 for early plots and 0.0013 for late plots.

**Table S1. Parameters and data sources for estimates of 2011 seed rain.**

| Parameter | Description | Estimate | Data source |
| --- | --- | --- | --- |
| *r* | mean number of racemes | 8.4368 for early  9.5432 for late | Mean across all plants in the demographic census in 2011 |
| *f* | mean number of fruits per raceme | 5.185 | Yarned racemes on 135 reproductive plants that produced dehisced fruits in 2011 |
| *s* | mean number of seeds per fruit | 3.376 | Mean seeds per fruit from 23, 3, 15, and 39 fruits collected in 2005, 2008, 2009, and 2010 |
| *c* | proportion of racemes consumed | 0.0973 | Yarned racemes on 148 reproductive plants in 2011 |
| *a* | proportion of racemes aborted | 0.1431 | Yarned racemes on 148 reproductive plants in 2011 |

**Table S2. Seedling establishment in 2012 in plots in early (E) and late (L) successional microhabitat.**

| Plot | Habitat  type | (1)  Number of  reproductive  plants in 2011 | (2)  Number of  seedlings  in 2012 | (3)  Estimated  seed rain  in 2011^$^ | (4)  Seedling  establishment  =(2)/(3) |
| --- | --- | --- | --- | --- | --- |
| SL-01 | E | 4 | 0 | 456.863 | 0 |
| SL-03 | E | 5 | 0 | 571.0787 | 0 |
| SL-05* | E | 1 | 2 | 114.2157 | 0.017511 |
| SL-07 | E | 5 | 4 | 571.0787 | 0.007004 |
| SL-09 | E | 3 | 7 | 342.6472 | 0.020429 |
| SL-11 | E | 2 | 6 | 228.4315 | 0.026266 |
| SL-13 | E | 2 | 2 | 228.4315 | 0.008755 |
| SL-15 | E | 7 | 2 | 799.5102 | 0.002502 |
| SL-17 | E | 8 | 4 | 913.7259 | 0.004378 |
| SL-19 | E | 6 | 10 | 685.2944 | 0.014592 |
| SL-02 | L | 2 | 0 | 258.3879 | 0 |
| SL-04 | L | 8 | 0 | 1033.552 | 0 |
| SL-06* | L | 5 | 1 | 645.9698 | 0.001548 |
| SL-08* | L | 6 | 3 | 775.1638 | 0.00387 |
| SL-10** | L | 4 | NA | 516.7759 | . |
| SL-12 | L | 3 | 0 | 387.5819 | 0 |
| SL-14* | L | 6 | 2 | 775.1638 | 0.00258 |
| SL-16 | L | 2 | 0 | 258.3879 | 0 |
| SL-18 | L | 4 | 2 | 516.7759 | 0.00387 |
| SL-20 | L | 7 | 0 | 904.3577 | 0 |

*$ Seed rain = (number of reproductive plants)rfs(1-c)(1-a)*

** Plot experienced some degree of sand burial due to sand blowing from restoration*

*** Plot experienced complete burial due to sand blowing from restoration*

***Recruitment of seeds from the seed bank***

To measure recruitment of seeds from the seed bank, we collected seeds of *L. tidestromii* (using mesh fabric to catch seeds from fruits) in 2008 and redistributed them directly back into seed baskets to measure recruitment in 2009, 2010, and 2011. As part of our larger study, we installed 6 seed baskets at Goat Rock State Beach (Sonoma County, CA) and Pacific Groves Golf Links (Monterey County, CA) and 12 seed baskets at Abbotts Lagoon (Marin County, CA) and Spanish Bay (Monterey County, CA). At Goat Rock and Pacific Groves, each seed basket was initiated with 50 seeds of *L. tidestromii*. At Abbotts Lagoon and Spanish Bay, six seed baskets contained 50 seeds of *L. chamissonis* and six baskets contained 50 seeds of *L. tidestromii*; additionally, three baskets of each species type also contained 50 seeds of a third co-occurring species, *L. arboreus*.

We constructed seed baskets by burying a 30 cm piece of 30 cm diameter corrugated plastic culvert 25 cm deep (leaving 5 cm exposed) in patches of sand with no immediately adjacent reproductive plants. We lined the interior of the culvert pipe with ¼-inch (0.64 cm) hardware cloth that extended 5 cm above the edge of the pipe. We sifted the sand inside the seed basket to remove other seeds and then placed seeds in the top 1 cm of sand. Seed baskets were covered with lids made of ¼-inch hardware cloth attached with plastic zipties. The hardware cloth prevented post-dispersal seed predation by mice. The raised lip of the culvert above the sand likely prevented some scarification of the seed but prevented loss from or addition of seeds to the basket from wind.

In June of each year we counted seedlings in each seed basket. We removed *L. chamissonis* and *L. arboreus* seedlings. For *L. tidestromii*, we did not pull seedlings in 2008 but in 2009 we discovered second-year plants could become reproductive and as seed addition would eliminate the basket from our analyses, we thus began removing germinated individuals of all species from all baskets each year.

Of 36 total seed baskets at all sites, 24 contained *L. tidestromii*. One seed basket at Spanish Bay was overturned and lost between 2008-2009 and 6 seed baskets at Goat Rock State Beach were removed in 2009 and thus yield only first year recruitment data; at Pacific Groves, two seed baskets had plants that germinated in 2008, were reproductive in 2009, and set seeds before the seedling census, and thus could not be used to estimate recruitment data for subsequent years. Data for all species and all baskets are provided in Table A3. For this analysis, we used 15 seed baskets containing *L. tidestromii* located at Abbotts Lagoon, Spanish Bay, and Pacific Groves that yielded recruitment data for three consecutive years (Table A3).

Mean recruitment for *L. tidestromii* after one winter was 0.0813 (range 0.00-0.24), after two winters (one year in the seed bank) was 0.0667 (range 0.00-0.32 among baskets), and after three winters (two years in the seed bank) was 0.0013 (range 0.00-0.02 among baskets) (Table A3, below).

***Estimating recruitment in early and late habitats over multiple years.***

To estimate recruitment of seeds in different microhabitats from the seedbank we adjusted the first year establishment rate (*e*_1_) using the estimates of recruitment from the seed bank. We calculated two constant values: *x*, the ratio of seeds that germinated after two winters (on year in the seed bank) to the number of seeds that germinated after one winter (i.e. 0.0667/0.0813=0.8197) and *y* the ratio of seeds that germinated after three winters (two years in the seed bank) to the number of seeds that germinated after one winter (i.e. 0.0013/0.0813=0.0164). The constants *x* and *y* do not vary across microhabitats or years.

Multiplying the establishment values from seedling plots (*e*_1_) by *x* and *y* allows our model to estimate habitat-specific recruitment from the seedbank. Further, recruitment in seedling plots included any loss of seeds to post-dispersal seed predation, which is a dynamic that was not captured by our exclosed seed baskets.

In our matrix population models, we assume that seeds do not persist in the seed bank for more than two years. This assumption matches our observations in the seed baskets. Seedling recruitment was observed in 2009-2011, but not in 2012. This assumption is reasonable in the current habitat, in which large disturbances that bring deeply buried seeds to the surface are nonexistent due to the stabilization of Abbotts Lagoon by European beachgrass. However, we note that *L. tidestromii* has a seed coat that should allow for long-term dormancy.

**Table S3. Seedling recruitment from the seed bank measured in seed baskets for *Lupinus tidestromii* (LT)*, L. chamissonis* (LC)*,* and *L. arboreus* (LA) at four sites.**

| ID | Site | Focal species | Species in the  seed basket | 2008 | 2009 | 2010 | 2011 | Used for  this analysis |
| --- | --- | --- | --- | --- | --- | --- | --- | --- |
| 68 | AL | LT | LT | 50 | 1 | 3 | 0 | * |
| 71 | AL | LT | LT | 50 | 11 | 0 | 0 | * |
| 79 | AL | LT | LT | 50 | 12 | 1 | 1 | * |
| 65 | AL | LT | LA+LT | 50 | 1 | 16 | 0 | * |
| 70 | AL | LT | LA+LT | 50 | 12 | 3 | 0 | * |
| 81 | AL | LT | LA+LT | 50 | 6 | 2 | 0 | * |
| 28 | GR | LT | LT | 50 | 5 | . | . |  |
| 29 | GR | LT | LT | 50 | 0 | . | . |  |
| 30 | GR | LT | LT | 50 | 6 | . | . |  |
| 31 | GR | LT | LT | 50 | 8 | . | . |  |
| 32 | GR | LT | LT | 50 | 4 | . | . |  |
| 33 | GR | LT | LT | 50 | 4 | . | . |  |
| 22 | PGR | LT | LT | 50 | 0 | 3 | 0 | * |
| 23 | PGR | LT | LT | 50 | 1 | 4 | 0 | * |
| 24 | PGR | LT | LT | 50 | 0 | 0 | 0 | * |
| 25 | PGR | LT | LT | 50 | 7 | 2 | . |  |
| 26 | PGR | LT | LT | 50 | 5 | 3 | . |  |
| 27 | PGR | LT | LT | 50 | 3 | 5 | 0 | * |
| 11 | SBY | LT | LT | 50 | . | . | . |  |
| 15 | SBY | LT | LT | 50 | 0 | 0 | 0 | * |
| 19 | SBY | LT | LT | 50 | 0 | 1 | 0 | * |
| 13 | SBY | LT | LA+LT | 50 | 0 | 5 | 0 | * |
| 17 | SBY | LT | LA+LT | 50 | 8 | 1 | 0 | * |
| 20 | SBY | LT | LA+LT | 50 | 6 | 6 | 0 | * |
| 66 | AL | LC | LC | 50 | 5 | 7 | 5 |  |
| 69 | AL | LC | LC | 50 | 4 | 4 | 0 |  |
| 80 | AL | LC | LC | 50 | 6 | 7 | 4 |  |
| 67 | AL | LC | LA+LC | 50 | 3 | 5 | 1 |  |
| 72 | AL | LC | LA+LC | 50 | 9 | 3 | 1 |  |
| 78 | AL | LC | LA+LC | 50 | 0 | 8 | 0 |  |
| 12 | SBY | LC | LC | 50 | 6 | 2 | 0 |  |
| 16 | SBY | LC | LC | 50 | 9 | 1 | 0 |  |
| 21 | SBY | LC | LC | 50 | 11 | 6 | 0 |  |
| 10 | SBY | LC | LA+LC | 50 | 17 | 7 | 0 |  |
| 14 | SBY | LC | LA+LC | 50 | 3 | 3 | 0 |  |
| 18 | SBY | LC | LA+LC | 50 | 8 | 4 | 0 |  |
| 65 | AL | LA | LA+LT | 50 | 3 | 9 | 5 |  |
| 70 | AL | LA | LA+LT | 50 | 20 | 8 | 2 |  |
| 81 | AL | LA | LA+LT | 50 | 21 | 0 | 1 |  |
| 67 | AL | LA | LA+LC | 50 | 8 | 8 | 1 |  |
| 72 | AL | LA | LA+LC | 50 | 8 | 8 | 2 |  |
| 78 | AL | LA | LA+LC | 50 | 2 | 5 | 0 |  |
| 13 | SBY | LA | LA+LT | 50 | 0 | 10 | 0 |  |
| 17 | SBY | LA | LA+LT | 50 | 0 | 2 | 0 |  |
| 20 | SBY | LA | LA+LT | 50 | 4 | 2 | 0 |  |
| 10 | SBY | LA | LA+LC | 50 | 2 | 4 | 0 |  |
| 14 | SBY | LA | LA+LC | 50 | 0 | 3 | 0 |  |
| 18 | SBY | LA | LA+LC | 50 | 0 | 4 | 0 |  |
| *Sites: AL=Abbotts Lagoon; GR=Goat Rock State Beach; PGR=Pacific Groves Golf Links; SBY=Spanish Bay* | | | | | | | | |
